# Perovskite Membranes: Advancements and Challenges in Gas Separation, Production, and Capture

**DOI:** 10.3390/membranes13070661

**Published:** 2023-07-12

**Authors:** Abdul Hai Alami, Adnan Alashkar, Mohammad Ali Abdelkareem, Hegazy Rezk, Mohd Shahbudin Masdar, Abdul Ghani Olabi

**Affiliations:** 1 Sustainable Energy & Power Systems Research Centre, University of Sharjah, Sharjah P.O. Box 27272, United Arab Emirates; aalalami@sharjah.ac.ae (A.H.A.); mabdulkareem@sharjah.ac.ae (M.A.A.); 2 Materials Science and Engineering Ph.D. Program, American University of Sharjah, Sharjah P.O. Box 26666, United Arab Emirates; b00028197@aus.edu; 3 Fuel Cell Institute, Universiti Kebangsaan Malaysia, Bangi 43600, Malaysia; 4 Department of Electrical Engineering, College of Engineering in Wadi Alddawasir, Prince Sattam Bin Abdulaziz University, Al-Kharj 11942, Saudi Arabia; 5 Mechanical Engineering and Design, School of Engineering and Applied Science, Aston University, Aston Triangle, Birmingham B4 7ET, UK

**Keywords:** perovskite membranes, hydrogen production, oxygen enrichment, carbon capture

## Abstract

Perovskite membranes have gained considerable attention in gas separation and production due to their unique properties such as high selectivity and permeability towards various gases. These membranes are composed of perovskite oxides, which have a crystalline structure that can be tailored to enhance gas separation performance. In oxygen enrichment, perovskite membranes are employed to separate oxygen from air, which is then utilized in a variety of applications such as combustion and medical devices. Moreover, perovskite membranes are investigated for carbon capture applications to reduce greenhouse gas emissions. Further, perovskite membranes are employed in hydrogen production, where they aid in the separation of hydrogen from other gases such as methane and carbon dioxide. This process is essential in the production of clean hydrogen fuel for various applications such as fuel cells and transportation. This paper provides a review on the utilization and role of perovskite membranes in various gas applications, including oxygen enrichment, carbon capture, and hydrogen production.

## 1. Introduction

Gas production and separation play an integral part in mitigating energy demands and supporting various industrial processes worldwide. The importance of efficient gas production and separation processes cannot be overstated, as they directly impact sectors such as power generation, fuel production, chemical manufacturing, and environmental sustainability [1]. Gas production involves the extraction and purification of gases from various sources, including natural gas fields, biogas plants, and industrial processes. The obtained gases often contain impurities or are in mixed compositions, requiring effective separation techniques to extract the desired components and remove contaminants.

Hydrogen (H_2_) gas provides a clean and sustainable fuel alternative to fossil fuels and natural gas [2]. Hydrogen production is generally performed through the methane stream reforming reaction, which leads to the by-product of carbon monoxide (CO) or carbon dioxide (CO_2_) in addition to H_2_ [3]. Another technique is the separation of H_2_ from steam either through pressure swing adsorption, cryogenic separation, and membrane separation [4]. The storage of hydrogen has also been investigated due to its crucial role in the hydrogen economy [5,6]. Membrane-based gas separation technology has become increasingly important in recent years due to its minimal energy consumption, cost-effectiveness, and scalability [7].

Membranes are utilized in a multitude of applications ranging from wastewater treatment [8] to pollutants removal and processing [9,10]. Membranes are utilized to separate different gases from mixtures based on their density, chemical properties, and concentration. There are several types of membranes used in gas production, including polymeric [11,12], ceramic [13,14], and metallic membranes [15,16]. Each type has its unique advantages and limitations in terms of selectivity, permeability, and cost. Perovskite-based membranes have emerged as a promising technology for gas production and separation due to their high selectivity and permeability towards certain gases such as H_2_, Oxygen (O_2_), and CO_2_ [17,18]. Perovskite materials have a unique structure that allows the efficient separation of gases based on their specific size and properties. In addition, Perovskite materials are recognized for their stability under harsh operating conditions, such as extreme temperature and elevated pressures, allowing a more durable and longer-lasting operation compared to other types of membranes. Scalability is another advantage of perovskite membranes, as the various fabrication techniques are compatible with large-scale operations.

In this paper, the utilization and role of perovskite membranes in gas production, separation, and capture applications is reviewed and presented. An outlook on the fabrication methods and deposition techniques of perovskite membranes is also summarized. This work highlights the advancement in perovskite membranes and provides a future outlook on their employment as a cost-effective and scalable method for hydrogen production, oxygen enrichment, and carbon dioxide capture.

## 2. Perovskite Membranes Structure and Fabrication Methods

**Figure 1:**
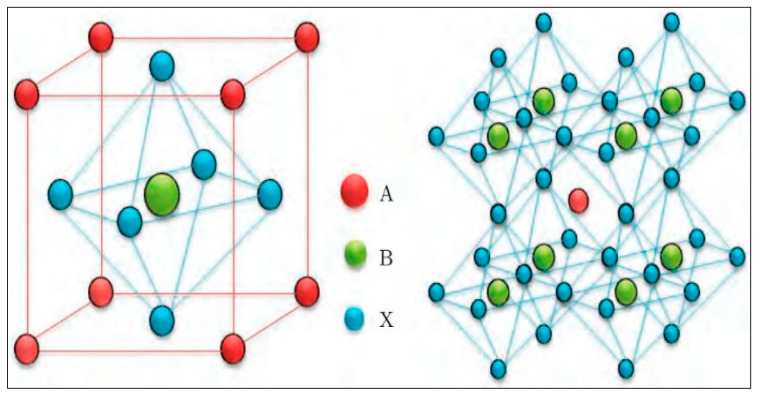
Perovskite Structure [26]. (Obtained from an open access source).

Perovskite materials have the unique cubic structure of ABX_3,_ where A and B are both positively charged ions (cations) that vary in size, and X is a negatively charged ion (anion). The structure is characterized by the cubic arrangement, where the larger A-site cations occupy the corner of the lattice, and the smaller B-site cations occupy the center of the lattice as shown in Figure 1. The X-site anions surround the B-site cation at the face-centered position. This arrangement allows the formation of a three-dimensional framework providing stability to the structure. The substitution of the X-site anion with an oxide (generally oxygen) results in a perovskite oxide material with the structure of ABO_3_. Both ABX_3_ and ABO_3_ perovskite structures have unique properties and can be used in various applications. For instance, ABX_3_ is mainly used in solar cells [19,20], where the A-site cation can be cesium (Cs^+^), methylammonium, or formamidinium; B-site cation can be lead (Pb^+2^), tin (Sn^+2^), or germanium (Ge^+4^); and the X-site anion can be either iodide (I^−^), chloride (Cl^−^), or bromide (Br^−^). Whereas ABO_3_ is mainly used as catalyst [21], with A-site cations varying between lanthanum (La), barium (Ba), and strontium (Sr). While B-site cations are transition metals such as molybdenum (Mo), tungsten (W), and zirconium (Zr). The X-site anion is typically an oxygen atom.

Perovskite materials have gained significant attention in various fields, including photovoltaics [22], optoelectronics [23], and catalysis [24], because of their tunable properties. Perovskite materials offer several distinct advantages compared to various membrane materials such as carbon materials, zeolites, MOFs (Metal–Organic Frameworks), and COFs (Covalent Organic Frameworks). For instance, due to the versatile structure of perovskite materials, a wide range of elements and compositions can be incorporated. This versatility enables the tailoring of material properties to suit specific applications, including tunable electronic, optical, and catalytic properties. Perovskite materials exhibit high ion conductivity, particularly in oxide perovskites. This property makes them suitable for various applications in oxygen separation membranes and gas sensors [25]. Their high ion transport capability enables efficient transport of ions through the material, enhancing the overall membrane performance.

Perovskite materials often exhibit excellent catalytic activity, especially in perovskite oxides. They can facilitate various catalytic reactions, such as oxygen reduction reactions (ORR), oxygen evolution reactions (OER), and hydrogen evolution reactions (HER). By adjusting the composition and structure, the bandgap of perovskite materials can be tuned over a wide range, allowing efficient absorption of sunlight across the solar spectrum. This tunability is advantageous for designing efficient and cost-effective solar cells [27]. Perovskite materials can be synthesized using solution-based techniques, which offer the advantage of low-cost and high-throughput production. Solution processing methods, such as spin coating and inkjet printing, enable large-area deposition of perovskite films, making them promising for scalable manufacturing [28]. Perovskite materials can be easily integrated with existing technologies and fabrication processes. This compatibility with established processes enhances their potential for commercialization and adoption. The complex synthesis procedure of MOFs and COFs presents a challenge for scalability as the high-cost limits large-scale industrial applications [29]. Zeolites have been widely utilized for large-scale applications. However, some zeolite types may require more complex synthesis methods or costly raw materials [30]. Table 1 provides a comparison summary of perovskite materials with various membrane materials.

The mechanical performance of perovskite membranes is a vital consideration for their industrial applications. Perovskite materials generally exhibit a degree of flexibility and elasticity, especially when they are fabricated as thin films or nanostructures [31]. This flexibility is advantageous in applications where the membrane needs to withstand bending or deformation without fracture or delamination. However, the specific mechanical properties can change based on the composition, structure, and processing conditions of the perovskite material [32]. Perovskite membranes are often deposited on supporting substrates to enhance their mechanical stability. The substrate can provide mechanical reinforcement and prevent the membrane from cracking or breaking under mechanical stress. The choice of substrate material and its compatibility with the perovskite membrane is vital in defining the overall mechanical performance and integrity of the membrane [33]. The interface between the perovskite membrane and the supporting substrate can significantly alter the mechanical properties. By optimizing the interfacial interactions and engineering suitable interlayers or adhesion promoters, it is possible to improve the adhesion and mechanical stability of the membrane. Various techniques, such as surface modifications and interfacial bonding strategies, can be employed to improve mechanical performance.

Perovskite membranes have the potential to be applied to various gas separation systems beyond H_2_, O_2_, and CO_2_ separation. The advantageous properties of perovskite materials, such as their tunable composition, high ionic conductivity, and selectivity, make them promising candidates for a multitude of gas separation applications. Perovskite membranes have the ability to be employed in natural gas processing for separating methane from other hydrocarbons or impurities [34]. Efficient methane separation is crucial for applications such as natural gas purification, upgrading, and transportation. Perovskite membranes with suitable composition and surface characteristics can enable the selective separation of methane from complex gas mixtures [35]. Perovskite membranes can be explored for the separation of various hydrocarbon gases, such as ethane, propane, and butane, from gas mixtures [36,37]. This has implications for applications like petrochemical processing and olefin/paraffin separations. The selectivity and transport properties of perovskite membranes can be tailored to achieve the desired separation performance. Perovskite membranes can potentially be used for the recovery and removal of volatile organic compounds (VOC) from gas streams. VOCs are present in industrial emissions and can have harmful environmental impacts. Perovskite membranes with high selectivity and permeability can facilitate the efficient removal and recovery of VOCs, aiding in pollution control and resource conservation [38].

The elemental composition of perovskite materials allows the multiple utilization of perovskite membranes in various gas separation processes. The separation mechanism of perovskite membranes is closely related to their elemental composition. The choice of elements in the perovskite structure directly impacts the membrane’s selectivity and transport properties. The elemental composition of perovskite, particularly the cationic species, influences the ionic conductivity of the membrane [39]. For example, the presence of mobile cations, such as alkali metals (e.g., K^+^, Na^+^), can enhance ionic conductivity by facilitating the movement of ions within the perovskite lattice. The elemental composition of perovskite affects its chemical affinity towards specific molecules or ions. This affinity can influence the selectivity of the membrane for certain components during separation processes. By selecting appropriate cations or anions in the perovskite structure, it is possible to tailor the adsorption and transport properties of the membrane, enabling the selective separation of desired species [40]. Perovskite membranes can exhibit mixed ionic-electronic conduction, where both ions and electrons contribute to the overall transport process. The elemental composition of the perovskite can influence the balance between ionic and electronic conduction, which impacts the membrane’s overall transport mechanism. For example, doping the perovskite structure with transition metal elements can enhance electronic conductivity, enabling efficient electron transport. The elemental composition of the perovskite affects its crystal structure and the presence of defects within the lattice. Structural defects, such as vacancies or substitutions, can impact the transport properties of the membrane [41]. For example, the introduction of dopant elements can create oxygen vacancies, which can enhance the oxygen ion transport in perovskite oxide membranes. The elemental composition of perovskite membranes influences their stability and chemical compatibility with the target separation environment. Certain elements may exhibit higher chemical stability, resistance to reactive species, or tolerance towards harsh operating conditions. By selecting appropriate elements in the perovskite composition, it is possible to improve the membrane’s durability and long-term performance.

In addition to the elemental compositions, the structure of perovskite membranes has a crucial effect on their performance. Various structural characteristics, such as composition, crystal structure, morphology, and defect chemistry, can significantly impact the membrane’s transport properties, selectivity, stability, and overall performance. The crystal structure of perovskite membranes affects their transport properties and stability. Variations in the crystal lattice parameters, such as lattice constant, bond lengths, and angles, can impact ion diffusion pathways, electronic band structure, and defect formation [42]. The crystal structure also determines the type of defects present in the membrane, which can influence the membrane’s ionic or electronic conductivity. The morphology of perovskite membranes, including grain size, porosity, and surface roughness, can influence their performance. Smaller grain sizes and higher porosity can enhance mass transport through the membrane by reducing diffusion pathways and providing larger surface areas for adsorption and reaction. Control over the membrane’s morphology can improve selectivity, flux, and stability. Defects in perovskite membranes, such as vacancies, substitutions, or interstitials, play a crucial role in their performance [43]. Defects can influence ionic or electronic conductivity, surface reactions, and chemical stability. The type, concentration, and distribution of defects can be tailored to optimize the membrane’s performance for specific applications, such as oxygen ion transport in solid oxide fuel cells or ion selectivity in ion exchange membranes. The interfaces of perovskite membranes, including the membrane–substrate interface and the membrane–environment interface, can significantly impact their performance [43]. Proper interface engineering can enhance adhesion, minimize interfacial reactions, and improve transport properties. The choice of interlayers or coatings, surface functionalization, or interface modification techniques can optimize the membrane’s performance and stability. The structural stability of perovskite membranes is crucial for long-term performance. Factors such as chemical compatibility, thermal stability, and resistance to reactive species or environmental conditions can affect the membrane’s durability and reliability. Optimizing the structural parameters, composition, and surface characteristics can enhance the membrane’s stability and extend its operational lifespan.

### 2.1. Perovskite Membrane Fabrication Methods

Perovskite membrane preparation follows a three-step process starting with powder synthesis and calcination, followed by processing to the desired geometry, and finally sintering. The sintering step is the most crucial in the preparation process as it determines the main features of the resulting membranes such as the porosity, grain shape and size, density, and the morphology of the surface. The most facile method of preparing perovskite membranes is by pressing powders in disks followed by sintering [44]. A well-established process for the manufacturing of thin films and dense membranes is the tape casting method. This method involves creating a slurry by dispersing inorganic powder in a solvent, which can be either water or organic liquids. The viscosity of the slurry controls the thickness of the resulting tape and can be adjusted by adding dispersing agents, binders, and plasticizers. The slurry is then applied onto a flat support to form a tape, where adjustable blades are employed to regulate the thickness of the membrane. For instance, La_0.6_Sr_0.4_Fe_0.9_Ga_0.1_O_3_ dense disk membranes with a thickness ranging from 0.63 to 1.30 mm [45]. Extrusion involves the passage of ceramic slurries through a die orifice subjected to high pressure. To achieve the desired shaping, the slurry must possess tailored rheologic properties. For instance, to be able to shape the geometry and press it through the die, a slurry with low viscosity is needed. On the other hand, a tube needs to maintain its annular shape, requiring a sufficiently high viscosity to reinforce the resulting geometry [46]. The shape of the materials is defined by the measurements and geometry of the die and the cutting length during the shaping process. Once the required shape is achieved, the processed material is subjected to a two-step sintering heat treatment. In the first step, the material is slowly heated to evaporate any organic compounds present in the extruded mixture. Following that, the material is sintered at elevated temperatures to ensure the densification of the membrane. This method has been utilized for the preparation of various perovskite membranes, such as SrCo_0.8_Fe_0.2_O_3_ and La_0.7_Ca_0.3_Fe_0.85_Co_0.15_O_3_ [47].

The process of producing perovskite hollow fibers starts by preparing a mixture called dope. In this method, precursor powders are combined with a solution that contains a polymer-based binder and a solvent. The mixture is carefully blended while being continuously stirred. It is essential to carefully regulate parameters such as the particle size of the powder, the ratio of solvent to binder, and the ratio of powder to binder in the final solution. Following the mixing step, the resulting dope solution is then spun to create a hollow fiber. Polyvinylpyrrolidone is commonly employed as an agent to adjust the viscosity in the production process. The dope solution, along with a liquid known as the bore liquid, is injected under pressure through a spinneret. This injection process gives rise to the formation of hollow fiber geometry. The completion of this geometry occurs after a coagulation bath, where the geometry contacts with a liquid bath. Several important parameters require careful control, including the viscosity of the dope solution, injection pressure, selection of bore liquid and coagulant liquid, as well as the air gap (i.e., the distance between the spinneret and the coagulant bath). Subsequently, the hollow fibers are subjected to high-temperature sintering (ranging from 410 to 1000 °C) under atmospheric conditions. During the sintering stage, the polymeric binder is burned off, transforming into carbon, as the polymers react with oxygen from the air at temperatures exceeding 300 °C, leading to the formation of CO_2_. As a result, the particles come into contact with each other and commence the formation of a perovskite material exhibiting a hollow fiber structure, commonly known as the green fiber.

The fabrication of perovskite thin films on porous substrates is a novel method that has gained major interest recently. However, most of the efforts have not yielded satisfactory outcomes yet. The commonly employed technique revolves around the concept of asymmetric membranes, where the porous substrate allows minimal resistance to gas molecule transport, while the thin film presents a higher resistance. It is understood that gas fluxes through membranes increase as the thickness decreases. This approach proves effective when the coefficient of expansion of the perovskite materials aligns with that of the substrate. Nevertheless, the challenge with perovskites lies in the fact that their coefficients of expansion are predominantly nonlinear [48]. The dip-coating method has been employed to deposit thin perovskite films on porous flat or tube substrates. However, this approach has resulted in ineffective membranes with significant defects that render them incapable of efficiently separating oxygen from the air. To address these challenges, substrates and films fabricated from the same perovskite material are utilized to match the coefficient of expansion between the layers. Nevertheless, even these perovskite membranes have failed to exhibit satisfactory oxygen separation capabilities. Recently, a novel approach has been investigated, involving the preparation of asymmetric films on porous substrates. The perovskite thin film is first prepared using the tape casting technique, followed by placing the porous substrate on top of it [49]. This technique yielded remarkable results for the fabrication of membranes from thin dense films of 70 μm and 20 μm, respectively [46,47,48,49,50].

Freeze-cast is another method utilized for the manufacturing of perovskite membranes. This technique shares similarities with the formation of dense films on porous substrates, where the substrates are fabricated through the Freeze-cast method. One significant benefit of Freeze-cast is the capability to produce mechanically stable and vastly porous substrates, where the pores possess organized channels. The method involves the preparation of a stable colloidal suspension, which is then poured into a mold. The suspension is then frozen, followed by the sublimation and sintering of the resulting material. The rapid freezing effect leads to the formation of a solidification front, effectively trapping perovskite particles within the growing crystals. During the sublimation of the slurry solvent/liquid, a structured network of pores is created and retained throughout the following high-temperature sintering processes. Water is commonly employed as a solvent in this environmentally friendly process. The morphology of the pores is influenced by the crystal growth of the solvent, necessitating precise control over the cooling rate [51]. Table 2 provides a summary of the advantages and drawbacks of the various perovskite membrane fabrication techniques.

### 2.2. Perovskite Membrane Deposition Methods

The deposition of perovskite on various membranes can be performed through several methods. The following section provides a summary of common perovskite membrane deposition techniques.

#### 2.2.1. Wet Chemical Methods

Wet chemical methods refer to the use of solution-based perovskite precursors that are then deposited on a substrate and transformed into perovskite thin films. The most widely used technique is spin coating. In this method, a solution containing perovskite precursors is spread onto a substrate, which is then rapidly rotated to achieve a uniform coating. Subsequent thermal annealing is usually performed to promote the formation of the perovskite phase [52]. Another technique is doctor blading, where a blade is used to spread the perovskite precursor solution onto a substrate. The excess solution is scraped off, leaving a thin, uniform film. This method is particularly useful for large-area deposition. Moreover, inkjet printing is a famous technique to deposit perovskite onto membrane substrates. The perovskite precursor solution is loaded into an ink cartridge, and droplets are selectively ejected onto specific locations to form the desired pattern. Inkjet printing is preferred due to the precise control of the thickness of the film thickness and the patterned deposition [52]. Similarly, slot-die coating involves the continuous deposition of a perovskite precursor solution through a narrow slit onto a moving substrate. The coating thickness is controlled by the gap between the slot die and the substrate, allowing uniform and scalable deposition [53]. Deposition techniques based on slurry have also been investigated. For instance, in slip casting, a mixture comprising fine powders with submicron dimensions, a liquid medium (such as ethanol or isopropanol), organic binders, and additives is formulated to achieve the desired flow characteristics, suspension stability, and sedimentation properties. When the mixture is poured onto a porous substrate, it permeates the pores of the support, resulting in the formation of a uniform and seamless layer of powder on the surface. The layer is then subjected to drying and subsequent sintering process [54]. On the other hand, in slurry spraying, powders suspended in a liquid are applied onto a substrate through the use of a spray gun. The resulting layer is then dried and subjected to sintering. This method is suitable for coating complex shapes; however, achieving precise control over the thickness at the edges of a component can be challenging. Another widely utilized technique is the sol–gel method. This process involves the immersion of a substrate in a solution followed by annealing. The thickness is controlled or modified by controlling the number of dips/annealing cycles. A sintering step is lastly conducted to achieve the required microstructure; hence, this method is particularly well-suited for creating such porous geometries [54].

#### 2.2.2. Plasma Spraying

The plasma spray technique involves the utilization of high DC power voltage electrodes to generate plasma. The particles are melted by the plasma and propelled at high velocities (ranging from 100 to 1200 m/s) through the plasma jet [55]. Upon contact with the substrate, the particles rapidly solidify, forming a deposition. The temperature of the plasma jet can vary significantly, ranging from 6727 to 19,727 °C [56]. Depending on the specific method employed, the powders can be deposited onto the substrate under different conditions: atmospheric pressure in the case of atmospheric plasma spray (APS), vacuum conditions (10^−2^ to 10^−3^ atm) in the case of vacuum plasma spray (VPS), and low pressure (<10^−3^ atm) in the low-pressure plasma spray method.

#### 2.2.3. Physical Vapor Deposition

Physical Vapor Deposition (PVD) techniques involve the vaporization of solid perovskite precursor material, followed by condensation on the substrate to form a perovskite thin film [57]. Some common PVD methods include thermal evaporation that requires precise control of temperature and pressure to accomplish the required film properties. The deposition is accomplished in a vacuum chamber. Electron Beam Evaporation (EBV) is another method, where an electron beam is utilized to vaporize the perovskite precursor material. The high-energy electron beam bombards the material, causing it to evaporate and subsequently deposit onto the substrate. This technique enables precise control over the deposition rate and allows the deposition of complex materials [58]. Sputtering is commonly used for deposition as well. Sputtering involves bombarding a material’s surface with high-energy ions, leading to the expulsion of atoms from the target. Subsequently, these expelled atoms settle on the substrate, creating a thin film. Both DC sputtering and magnetron sputtering can be used for perovskite membrane deposition [59]. Recently, Molecular Beam Epitaxy (MBE) has been used as a PVD technique. MBE is a precise deposition technique used for growing high-quality thin films. It involves the evaporation of constituent elements or compounds in an ultra-high vacuum environment, where the vaporized species condense onto the substrate with accurate control over the composition and thickness of the resulting film. MBE is commonly used for the epitaxial growth of perovskite films [60]. Generally, PVD techniques offer control over film thickness, composition, and crystallinity, making them suitable for the fabrication of perovskite membranes with tailored properties. However, it is worth noting that the application of PVD techniques for perovskite membranes may require specific adaptations and optimizations due to the sensitivity of perovskite materials to high temperatures and reactive environments.

#### 2.2.4. Chemical Vapor Deposition

**Figure 2:**
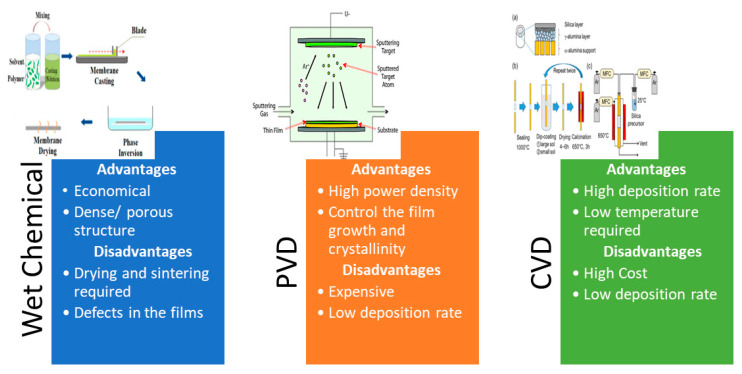
Advantages and drawbacks of various perovskite deposition techniques.

Chemical Vapor Deposition (CVD) techniques can be employed to fabricate perovskite membranes. In a CVD process, a perovskite precursor with a low vaporization point is usually heated to form active gas phases. The active gas then reacts to form the thin film on the substrate in a vacuum-controlled space referred to as the reaction chamber. Several CVD techniques can be used for the deposition of perovskite materials. For instance, in Atmospheric Pressure Chemical Vapor Deposition (APCVD), the perovskite precursor gases are introduced into a reactor chamber at atmospheric pressure. The substrate, usually a porous support material, is annealed to a certain temperature that allows the precursor gases to react and form a perovskite film. In addition, Low-Pressure Chemical Vapor Deposition (LPCVD) involves operating the reactor at reduced pressures, typically below atmospheric pressure. The lower pressure helps in achieving better control over film growth and allows the use of lower temperatures. The perovskite precursors are added into the chamber, where they decompose and react to form the desired perovskite membrane. Metal–Organic Chemical Vapor Deposition (MOCVD) utilizes metal–organic precursors, such as metal halides or metal alkoxides, to deposit perovskite films. The precursors are introduced into the reactor chamber along with a carrier gas, and they react at elevated temperatures to form the perovskite structure. MOCVD offers precise control over the film’s thickness and morphology. Plasma-Enhanced Chemical Vapor Deposition (PECVD) utilizes the use of plasma to enhance the deposition process. The perovskite precursors are introduced into a reactor chamber, and plasma is generated either by applying an electric field or by using microwave energy. Plasma helps in dissociating and activating the precursor molecules, allowing the deposition of perovskite films. CVD techniques provide flexibility in tailoring the composition, morphology, and thickness of perovskite membranes. The specific deposition parameters, such as temperature, pressure, precursor flow rates, and reaction time, need to be optimized to achieve high-quality perovskite films with desired properties. Additionally, post-deposition treatments like annealing or surface modifications may be applied to further enhance the membrane performance. Figure 2 shows the advantages and drawbacks of various perovskite deposition techniques.

## 3. Perovskite Membranes in Hydrogen Production

Hydrogen production through perovskite membranes is conducted with the aid of permeation through three sequential steps. First, a gas–solid exchange process occurs at the interface upstream of the membrane surface side, and then, bulk diffusion of the proton through the membrane takes place. Subsequently, a second surface exchange at the gas–solid interface downstream of the membrane occurs. These surface exchanges generally involve the interaction between the oxygen vacancies and oxygen lattice with dry and wet hydrogen according to the following reactions [61].

(1) $$H_{2}O+V_{\ddot{o}}+O_{O}^{x}\;↔2{\mathrm{OH}}_{\;\overset{\text{˙}}{O}}$$

(2) $$O_{O}^{x}+\frac{1}{2}H_{2}↔{\;\mathrm{OH}}_{\;\overset{\text{˙}}{O}}+\;e\;\text{′}$$

where ${\mathrm{OH}}_{\;\overset{\text{˙}}{O}}$ is a hydroxyl ion associated with a lattice of oxygen $O_{O}^{x}$, and $V_{\ddot{o}}$ is an oxygen vacancy.

**Figure 3:**
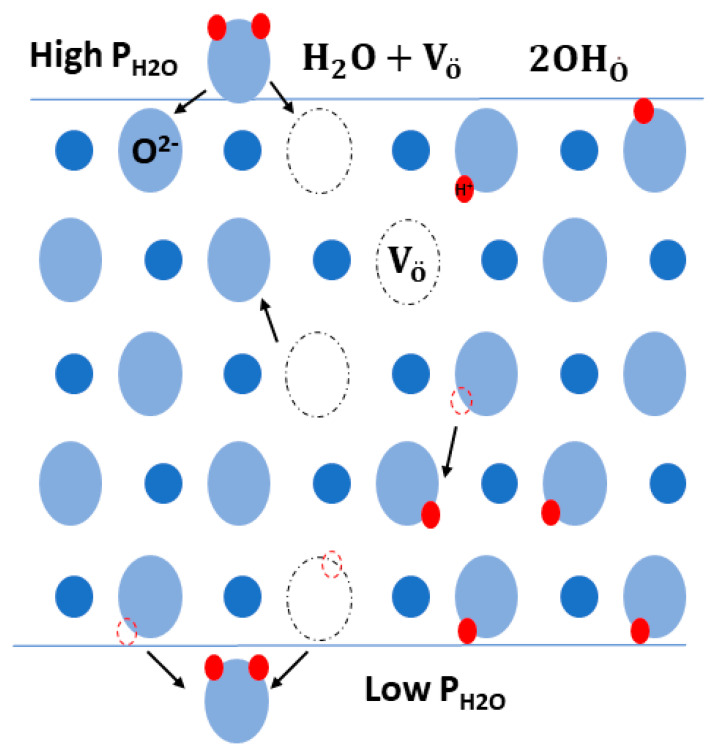
Proton transport mechanism.

Generally, it is believed that the proton diffusion through perovskite membranes is conducted through either vehicle or Grotthuss mechanisms [62]. The vehicle mechanism is named as such because the proton ($H^{+})$ attaches to the oxygen ions to form ${\mathrm{OH}}^{-}$ rather than moving individually. In the Grotthuss mechanism, the diffusion of the protons is conducted through bouncing between the adjacent lattice oxygen. In order to achieve this movement, first the redirection of the hydroxide ion occurs followed by a proton transfer between the oxygen ions. Thus, in the Grotthuss mechanism, the proton is the only mobile species, whereas the oxygen is localized in the vicinity of the crystallographic position. Recent studies have displayed that the Grotthuss mechanism is the main mechanism behind the diffusion of protons in perovskite oxide membranes [63]. Figure 3 shows the proton transport mechanism.

### 3.1. Improvements in Transport Mechanism

Non-doped perovskite oxide membranes are usually characterized by their poor proton and electron conductivities. Nevertheless, the presence of hydrogen increases the protonic conductivity of the perovskite oxide material due to the defect reaction between the hydrogen and the oxygen vacancy. A facile yet effective method to increase the protonic and electronic conductivities and in turn increase the hydrogen permeability of perovskite oxide materials is through the utilization of dopants. Typically, non-doped materials have a low concentration of inherent flaws, resulting in low levels of ionic conductivity in most situations. The defect concentration of proton conductors can be adjusted through aliovalent doping, specifically acceptor doping, in turn improving the ionic conductivity. By introducing acceptors, the number of oxygen vacancies can be increased, thereby enhancing the conductivity of oxide ions. Additionally, the proton conductivity is also enhanced through the hydration of these vacancies in wet H_2_ gas environment. The number of oxygen vacancies continues to increase with the increase in the dopant concentrations until the solubility limit of the dopant is reached. However, beyond a certain threshold of dopant concentration, a phenomenon known as defect associations might be presented. This phenomenon is a result of the interactions between different defects and leads to the leveling off or deterioration in conductivity despite the increase in dopant concentration [64].

Perovskite materials with the structure of BaCeO_3_, BaZrO_3_, SrCeO_3_, and SrZrO_3_ have the highest proton conductivities; thus, most research has been fixated on such materials [22,23]. BaCeO_3_-based perovskite oxide materials record the maximum protonic conductivity of 10^−2^ S cm^−1^ at 700 °C, yet their decomposition when subjected CO_2_ and humid atmosphere poses a serious limitation in their application. A method to increase the proton conductivity is the doping of Ce with cations possessing a larger ionic radius or with dopants that have a higher electronegativity [65]. In that regard, yttrium (Y) is the most successful co-dopant to create a mixed protonic electrical conductivity. On the hand, ionic-electronic conductivity can be created through doping with ytterbium (Yb) or praseodymium (Pr). This relation is accredited to the ionic radius and electronegativity of the ions, where yttrium lies in close proximity to Ce in terms of the ionic radius and possesses a much higher electronegativity, yielding an ideal B-site dopant. Whereas the significantly larger ionic radius of Pr and the much smaller ionic radius of Yb (both compared to Ce) results in unsuccessful doping [66,67]. The summation of the ionic and electronic conductivities (i.e., electrical conductivity), rises with the reduction in the ionization potential. Hence, the increase in electronic conductivity yields an improvement in the H_2_ permeation flux [22]. The operational conditions also influence the performance of perovskite membranes. For instance, utilizing N_2_ or O_2_ as sweep gas yielded a SrCe_0.95_Tb_0.05_$O_{3-\delta }$ membrane impermeable to hydrogen due to the lower electrical conductivity. Nevertheless, substituting the sweep gas with a reducing CO gas increased the electronic conductivity and allowed the membrane to become hydrogen permeable [68].

### 3.2. Disk-Shaped Membranes

Disk-shaped membranes are the most facile to prepare and are the most researched compared to their counterparts. These membranes can be categorized as single-phase or bi-phase membranes, where two individual composite membranes are stacked together. Even since Iwahar et al. [69] revealed the elevated conductivity of SrCeO_3_ perovskite material in hydrogen-enriched environment, their utilization as hydrogen separation membranes has been vastly investigated. Thereafter, BaceO_3_ perovskite membranes were studied, but they showed high oxygen ionic conductivity, which limited their use in oxygen-enriched atmospheres [70]. Thus, doping SrCeO_3_ to improve their electronic conductivity became an interest in hydrogen production membranes. The introduction of terbium (Tb) as a B-site dopant for perovskite material with SrCeO_3_ structure adversely affected the electron conductivity of the material in both air and inert gas. The hydrogen permeation fluxes of such materials were tested under various sweep gases, and the results showed that negligible detection was recorded in argon (Ar) or even 20% vol O_2_ in Ar. Nevertheless, by introducing small amounts of CO reducing gas (around 1 vol%) in an Ar-enriched environment (99 vol%), hydrogen flux is measured [68].

The doping of the B-site of SrCeO_3_ with Zr has also been examined. Kniep and Lin [61] reported on the performance of SrCe_0.95_Tm_0.05_$O_{3-\delta }$ perovskite material doped with Zr to form SrCe_0.95−x_Zr_x_Tm_0.05_$O_{3-\delta }$. The results displayed that higher Zr content hinders the electronic conductivity of the doped perovskite material due to the low conductivity of zirconates. Moreover, raising the Zr concentration adversely affected the protonic conductivity of the perovskite material. Thus, the hydrogen permeation decreased with the increase in Zr content. In a CO_2_ environment, the H_2_ permeation flux and chemical stability of SrCe_0.95−x_Zr_x_Tm_0.05_$O_{3-\delta }$ were superior to those of SrCe_0.95_Tm_0.05_$O_{3-\delta }$. To increase the resistance of perovskite membranes to CO_2_, doping the B-site with indium (In) is investigated. For instance, doping SrCe_0.95_Tm_0.05_$O_{3-\delta }$ with In to produce SrCe_0.95−x_In_x_Tm_0.05_$O_{3-\delta }$ improved the resistance of the membrane to CO_2_, and the increase was directly proportional to the In content. Nevertheless, the presence of In deteriorated the protonic conductivity and caused a decrease in the hydrogen permeation as compared to the non-doped perovskite membrane [71]. Fe-doping also proved to be an efficient method to improve the H_2_ flux of perovskite membranes. BaZr_0.9_Fe_0.1_$O_{3-\delta }$ membranes reported an improved H_2_ permeation flux of 0.75 mL cm^−2^ min^−1^ at 900 °C under 20 vol% H_2_ in Ar sweep gas [72]. The enhancement was attributed to the improvement in electron conductivity due to the valence changes from the Fe^2+^/Fe^3+^ pair. Moreover, a decrease in the membrane thickness increased the hydrogen permeability insinuating that the permeation is constrained by bulk diffusion. Another study investigated the H_2_ permeation of BaCe_0.95_Nd_0.05_$O_{3-\delta }$ membranes under wet and dry conditions and revealed that the greatest flux of 0.02 mL cm^−2^ min^−1^ occurred when the membrane was subjected to wet conditions [73]. Table 3 summarizes the performance of single-phase disk-shaped perovskite membranes in terms of hydrogen permeation flux. The apparent trend is that the H_2_ flux decreases with the increase in the membrane’s thickness. The testing temperature range of the membranes was in the range of 900 °C.

Although doping led to improvement in the hydrogen permeation of perovskite membranes, the process is not capable of producing materials that exhibit high protonic and electronic conductivities simultaneously. A different approach to address this problem involves the mixture of two distinct materials: one with high electronic conductivity and one with high proton conductivity. This combination leads to the formation of composite membranes, often referred to as bi-phase membranes, such as cermet (ceramic–metallic) and cercer (ceramic–ceramic) membranes. By incorporating a metallic phase, the electronic conductivity is increased, and the catalytic reactions between the layers of the membranes are improved. While the ceramic phase improves the mechanical strength of the composite membrane. Various combinations can be achieved based on the hydrogen permeability of both the ceramic and metallic phases. These combinations include: (i) pairing a metal/alloy with high permeability to a ceramic with low permeability, (ii) pairing low permeability metal with a high permeability ceramic, or (iii) combining a metal/ alloy with a ceramic where both have high permeability.

Membranes processed through the first combination (i.e., high-hydrogen permeability metal with low-hydrogen permeability ceramic), especially those processed with palladium (Pd), recorded the highest hydrogen flux. For instance, a permeation flux of 1.25 mL cm^−2^min^−1^ was recorded for a Pd-BaCe_0.4_Zr_0.4_Gd_0.1_Dy_0.1_$O_{3-\delta }$ in a mixture of 50 vol% H_2_ and 50 vol% CO_2_ mixture [78]. Nevertheless, given the expensive cost of Pd, alternative metals such as nickel have been investigated especially in membranes processed through the second combination (i.e., low-hydrogen permeability metal with high-hydrogen permeability ceramic). For instance, an H_2_ permeation flux of 0.76 mL cm^−2^min^−1^ was measured for a 230 μm thick Ni-BaZr_0.7_Ce_0.9_Y_0.1_$O_{3-\delta }$ perovskite membrane using a mixture of H_2_ and He sweep gas. Another perovskite membrane with structure Ni-BaCe_0.7_Y_0.2_$O_{3-\delta }$ in the same conditions achieved a flux of 0.805 mL cm^−2^min^−1^ [79]. Moreover, the hydrogen flux under wet conditions was higher by four and three folds compared to dry conditions at temperatures of 600 °C and 900 °C, respectively. This increase is attributed to the infiltration of moisture into the ceramic phase, increasing its proton conductivity. Copper (Cu) is another metal candidate that has been explored especially in carbon-containing atmospheres. Although Cu-based membranes such as Cu-BaZr_0.9_Y_0.1_$O_{3-\delta }$ recorded a substantially lower hydrogen flux of 4.6 × 10^−4^ mL cm^−2^min^−1^_,_ they did display excellent stability and remained leak-free for 30 days [80].

Membranes prepared through the third combination (i.e., high-hydrogen permeability metal with high-hydrogen permeability ceramic), have not yielded satisfactory hydrogen flux ratios due to issues at the interfacial layer between the metal and ceramic phases. Generally, atomic diffusion in the metallic phase, rather than ambipolar diffusion, is the source of hydrogen transport through cermat membranes. Thus, the main reason for the low performance of the microstructure can be accredited to the limitations in the ambipolar diffusion [81]. Theoretically, perovskite-based materials that conduct protons can also possess electronic conductivity when the crystal lattice position known as B-site is occupied by transition metal cations. However, the electronic conductivity of these materials is normally less than their ionic conductivity. To further enhance the electronic conductivity in such cases, the addition of a second polycrystalline ceramic phase with electronic conductivity can be beneficial. It is important, though, that these two multicrystalline ceramic phases construct a composite structure called cercer, where there is a sufficient level of percolation. This ensures the presence of continuous pathways for both proton and electron transport throughout the layers of the membrane. Thermal and chemical compatibility between the two multicrystalline phases is vital as membranes will be subjected to environments with high temperature reduction gases. For instance, the hydrogen flux was improved to 0.039 mL cm^−2^min^−1^ with the addition of ZnO to SrCe_0.95_Y_0.05_$O_{3-\delta }$ perovskite membrane [82]. H_2_ flux of 0.0107 mL cm^−2^min^−1^ was reported for a dual phase of 1.44 mm thick membrane with the structure BaCe_0.8_Y_0.2_$O_{3-\delta }$-Ce_0.8_Y_0.2_$O_{2-\delta }$ [83]. The preparation of a dual phase 370 μm La_5.5_W$O_{11.25-\delta }$-La_0.87_Sr_0.13_Cr$O_{3-\delta }$ membrane resulted in a H_2_ flux in the range of 0.15 mL cm^−2^min^−1^ by overcoming the low electronic conductivity and low sinterability of La_5.5_W$O_{11.25-\delta }$ and La_0.87_Sr_0.13_Cr$O_{3-\delta }$, respectively [84]. Table 4 provides a summary of the hydrogen permeation flux of cermat and cercer dual-phase disk-shaped perovskite membranes. The testing temperature range of the membranes was between 700–900 °C. As seen from the table, the H_2_ flux is influenced by the ratio of phases, the sweep atmosphere, and the thickness of the membranes. Cermat membranes prepared through the first combination (especially with Pd) showed the highest hydrogen permeation flux compared to their cermat counterparts and to cercer perovskite membranes in general. Moreover, thin membranes provided better hydrogen permeation flux when compared to thicker membranes.

### 3.3. Asymmetric Membranes

As perovskite membranes become thin enough, the overall flow of hydrogen does not solely depend on the diffusion rate of the protons through the membrane but also depends on the surface reactions. These surface reactions are influenced by the concentration of the boundaries, which are the boundaries between the phase that conducts protons, the phase that conducts electrons, and the gas phase. In order to overcome the thickness limitation of relatively thick disk membranes while maintaining mechanical integrity, an asymmetric structure is used. This structure combines a porous substrate/electrode with a thin perovskite layer. The porous layer contributes towards the mechanical stability of the membrane and allows gas to pass through its channels. Moreover, the thermal expansion of the porous and dense perovskite layers should match in order to avoid structural cracking throughout the sintering process. To ensure compatibility between the layers, similar materials are typically used for both the porous and perovskite layers. It is also important to select a suitable porous substrate that permits acceptable gas diffusion to facilitate the transport of hydrogen to the perovskite layer. As discussed in the previous sections (Section 2.1 and Section 2.2), the fabrication technique of the membrane and the deposition method of the perovskite material has an integral part in optimizing the conductivities and in turn the hydrogen permeation flux. For instance, perovskite layers of BaCe_0.9_Y_0.1_$O_{3-\delta }$ and BaCe_0.9_Y_0.1_$O_{3-\delta }$-rapidly solidified Zr were deposited by aerosol deposition on top of a porous ZrO_2_ desk [93]. The resulting membranes recorded a hydrogen flux of 0.113- and 0.17-mL cm^−2^min^−1^_,_ respectively. In addition, an asymmetric membrane BaCe_0.85_Tb_0.05_Zr_0.1_$O_{3-\delta }$ prepared through dry pressing revealed a H_2_ permeation flux of 0.07 mL cm^−2^min^−1^ stable over a period of 370 h [94]. Another asymmetric membrane prepared by depositing SrCe_0.95_Yb_0.05_$O_{3-\delta }$ on SrCe_0.95_Y_0.05_$O_{3-\delta }$ porous substrate reported a hydrogen permeation flux 500 times greater than symmetric SrCe_0.95_Y_0.05_$O_{3-\delta }$ membranes [95] Preparation of asymmetric membranes consisting of a dense SrCe_0.7_Zr_0.2_Eu_0.1_$O_{3-\delta }$ perovskite material on a porous Ni-SrCe_0.8_Zr_0.2_$O_{3-\delta }$ substrate resulted in a hydrogen permeation flux of 0.21–0.23 mL cm^−2^min^−1^ depending on the feed gas [96].

Tape casting was employed to construct an asymmetric Sr(Ce_0.6_Zr_0.4_)_0.85_Y_0.15_$O_{3-\delta }$ membrane by taping and pressing 10 layers of NiO/Sr(Ce_0.6_Zr_0.4_)_0.85_Y_0.15_$O_{3-\delta }$ and one layer Sr(Ce_0.6_Zr_0.4_)_0.85_Y_0.15_$O_{3-\delta }$ [97]. The membrane recorded an outstanding H_2_ flux of 4.12 mL cm^−2^min^−1^. Asymmetric Ni-BaCe_0.95_Tb_0.05_$O_{3-\delta }$ membranes prepared through dry pressing revealed a flux of 0.914 mL cm^−2^min^−1^ exhibited twice the flux of a desk-shaped Ni-BaCe_0.95_Tb_0.05_$O_{3-\delta }$ membrane [98]. Particle suspension coating was employed to fabricate a Ni-BaZr_0.1_Ce_0.7_Y_0.1_Yb_0.1_$O_{3-\delta }$ membrane supported on a porous Ni-BaZr_0.1_Ce_0.7_Y_0.1_Yb_0.1_$O_{3-\delta }$ substrate [99]. The asymmetric membrane displayed a hydrogen permeation flux of 0.49 mL cm^−2^min^−1^ and 1.12 mL cm^−2^min^−1^ at 700 °C and 900 °C, respectively. Table 5 summarizes the H_2_ permeation flux performance of asymmetric-shaped perovskite membranes.

### 3.4. Hallow Fiber Membranes

Hollow fiber membranes with a distinct asymmetric structure can be synthesized based on a specific inversion process. This structure consists of either a thin layer positioned on a porous layer, or a thin layer sandwiched in the middle of dual porous layers. Generally, all the layers are fabricated from the same material and through a single process, thus making the synthesis advantageous by eliminating any concerns related to thermomechanical compatibility. The hollow fiber configuration offers notable benefits for industrial applications, including low transport resistance due to the thin layer and a significant surface area per unit volume. However, the main challenge currently is to enhance the mechanical performance of the hollow fiber membranes. One strategy to overcome this issue is the use of hallow fiber bundles or a multi-bore structure. The first hollow fiber membrane SrCe_0.95_Yb_0.05_$O_{3-\delta }$ was prepared by Li et al. [102] and reported a H_2_ flux of 0.2 mL cm^−2^min^−1^ at 950 °C. Tan et al. [86] showed that the H_2_ flux can be improved by transforming disk membranes into hollow fiber membranes. For instance, the H_2_ flux rose from 0.01to 0.38 mL cm^−2^min^−1^ when BaCe_0.8_Yb_0.2_$O_{3-\delta }$ membrane was structured as a hollow fiber as compared to a disk membrane. Another study investigated the effect of doping on hollow fiber membranes and concluded that doping does not necessarily improve the hydromel permeability of hollow fiber membranes. For instance, doping BaCe_0.95_Tb_0.05_$O_{3-\delta }$ with Co revealed a lower hydrogen flux of around 0.19 mL cm^−2^min^−1^ [103].

Surface modification of hollow fiber membranes can be utilized to improve the permeation flux. Polishing the membrane will increase the surface roughness and, in turn, increase the effective surface area of the membrane. For example, NiO-coated BaCe_0.95_Tb_0.05_$O_{3-\delta }$ hollow fiber membrane recorded a H_2_ flux of 0.53 mL cm^−2^min^−1^ [104]. The hydrogen permeation performance can be improved by enhancing the surface kinetics of the membrane. This can be accomplished by depositing catalysts such as nickel and palladium. Coating a BaCe_0.85_Tb_0.05_Co_0.1_$O_{3-\delta }$ hollow fiber membrane with Pd and Ni increased the hydrogen permeation flux by 0.256- and 0.105-mL cm^−2^min^−1^, respectively [105]. Surface modification by H_2_SO_4_ etching and Pd coating enhanced the hydrogen flux of BaCe_0.95_Tb_0.05_$O_{3-\delta }$ hollow fiber membranes from 0.044 to 0.158- and 0.21-mL cm^−2^min^−1^_,_ respectively [106]. Nevertheless, the hollow fiber membrane coated with H_2_SO_4_ deteriorated with time. Table 6 summarizes the H_2_ flux performance of hollow fiber-shaped perovskite membranes.

## 4. Perovskite Membranes in Oxygen Separation

Oxygen is widely used in modern industrial processes and is considered one of the most commonly utilized gases. However, producing pure oxygen on a large scale is expensive and challenging, often requiring energy-intensive steps. As a result, many processes rely on air as the primary source of oxygen. Nevertheless, producing oxygen from atmospheric air is associated with the production of several other gases that need to be handled and efficiently stored. 

Oxygen Transport Membranes (OTMs) provide a novel and evolving technique to extract high-purity oxygen (approximately 99%) from the atmosphere. In this method, a chemical or electrical force is applied to the dense ceramic membranes in order to facilitate the diffusion of oxygen as ions. When compared to cryogenic and pressure swing methods, the utilization of OTMs for oxygen production offers several distinct advantages: (i) a cost reduction of approximately 40% in oxygen generation compared to existing technologies, (ii) enhanced efficiency in the process, (iii) reduced power consumption, and (iv) the ability to be utilized as a waste heat recovery system when added to power generation units [108,109]. Furthermore, when incorporated into a power cycle, the ceramic membrane technique eliminates the need for high-temperature heating and pressurization of the oxygen gas stream, which is typically required in cryogenic methods. Perovskite materials, classified as mixed (oxygen) ionic–electronic conductors (MIECs), have garnered considerable interest as suitable materials for OTMs [25,110,111]. These MIECs possess the ability to conduct both electrons and oxygen ions. When employed as membranes subjected to different atmospheres with fluctuating oxygen potentials, they enable the transportation of oxygen through the potential gradient. Utilizing perovskite-based oxygen separation membrane technology proposes several advantages, particularly in integrating it into power generation cycles that involve capturing CO_2_ through oxy-fuel combustion.

### 4.1. Oxygen Transport Mechanism

OTMs can be categorized as passive or active membranes depending on the driving force powering the oxygen transport mechanism. In passive membranes, an electrical potential is applied to facilitate the diffusion of oxygen ions through the perovskite membranes. This approach is cost-effective and reliable for generating high-purity oxygen while maintaining precise control over the volume of oxygen produced [22]. To maintain charge neutrality, an external electrical circuit and power source are needed to facilitate the movement of electrons across the membrane, hence, the designation of “electrically driven” membranes. The electro-neutrality is maintained through the following reaction:

(3) $$O_{o}^{\times }↔\frac{1}{2}O_{2}\left(g\right)+V_{o}^{\cdot \cdot }+2e^{-}$$

where $V_{o}^{\cdot \cdot }$ and $e^{-}$ refer to the oxygen and electron vacancy, respectively. While the symbols × and · refer to the neutral and positive charge, respectively.

In the active membrane driven by oxygen partial pressure, the dense perovskite material used for oxygen separation functions as an MIEC. This class of membranes operate based on the existence of a differential in oxygen partial pressure between an oxidizing environment (such as air at 0.21 atm) and a reducing gas (such as fuel at 10^22^ atm) [30]. This differential serves as the driving force for oxygen separation. It enables the transportation of O_2_ ions from the surface with high pressure to the low-pressure surface. Simultaneously, electrons move from the low to the high-pressure surface, completing the internal electric circuit. Consequently, an external circuit or power supply is not required.

**Figure 4:**
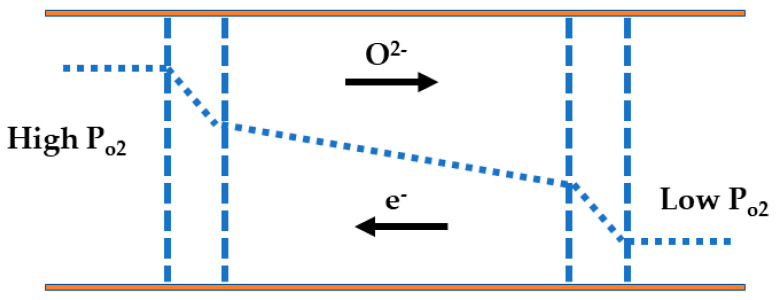
Representation of oxygen permeation process through an MIEC.

The process of oxygen permeation through an MIEC membrane involves a series of steps in sequence: (1) diffusion of oxygen molecules from the gas stream to the surface of the membrane, (2) reaction between oxygen molecules and vacancies at the membrane surface, (3) bulk diffusion of oxygen ions (or vacancies) through the membrane, (4) reaction between oxygen and electron-hole at the membrane surface, and (5) mass transfer of oxygen from the membrane surface back to the gas stream. Figure 4 shows the steps involved in the oxygen permeation process through an MIEC. The process of oxygen permeation through a membrane primarily relies on two factors: surface/interfacial exchange and bulk diffusion within the membrane. In order to relate both transport mechanisms, a characteristic thickness value, denoted as Lc, is introduced. This ratio is calculated by dividing the oxygen self-diffusivity by the surface exchange coefficient [112]. Membranes with a thickness lower than L_c_ show better O_2_ permeation. Once the thickness goes below Lc, further reduction does not improve the oxygen flux. However, it is possible to significantly increase the oxygen flux by depositing a MIEC layer (acting as a surface exchange and intermediate layer) onto the membrane. This deposition enhances surface kinetics and promotes efficient oxygen exchange at the interlayers [112]. The determining factor for the rate-limiting step in oxygen transport relies on a number of factors such as membrane thickness, operating temperature, and the gradient of oxygen partial pressure. When the membrane is thin, the dominant step in oxygen transport is the surface exchange reaction. However, for thicker membranes, diffusion becomes the dominating factor [112,113]. For instance, oxygen ions are first adsorbed at the surface before travelling through the bulk and through oxygen vacancies. The permeation flux of O_2_ enhances as the differential P_o2_ (gradient of oxygen chemical potential) and temperature increase, primarily due to a decrease in the energy required for oxygen ion migration [112,113]. It is worth noting that in dense membranes processing defects may result in the presence of closed pores. This results in O_2_ diffusion through the pores and the formation of a new path.

### 4.2. Improvements in Perovskite Oxygen Transport Membranes

Perovskite materials for MIEC were reported by Teraoka et al. [114], where perovskite oxide La_1−x_Sr_x_Co_1−y_Fe_y_$O_{3-\delta }$ materials displayed high electrical conductivity and oxygen permeability. Based on these findings, future work focused on the replacement of A and B ions in the mixed conducting oxide La_1−x_Sr_x_Co_1−y_Fe_y_$O_{3-\delta }$ [115]. The findings indicated that the order of oxygen permeation improvement follows Na < Sr < Ca < Ba (with Na, Ca, and Ba replacing Sr in the A site) and La < Pr < Nd < Sm < Gd (with Pr, Nd, Sm, and Gd substituting La in the A site). Furthermore, it was observed that permeation is increased with the increase in O_2_ vacancies, which can be accomplished by adding Cu and Ni cations into the B-site of the perovskite structure. Conversely, doping with Fe, Cr, and Mn in the B site adversely affected O_2_ permeation flux. This outcome suggests that the partial substitution of A and B cations significantly influences the oxygen permeation performance due to changes in their composition and phase structure. It gradually became apparent that the fabricating of stable perovskite OTM is as much a priority as enhancing oxygen permeation. For instance, OTM based on SrCr_0.8_Fe_0.2_$O_{3-\delta }$ displayed outstanding O_2_ permeability reaching 2.82 mL cm^−2^ min^−1^ at a temperature of 850 °C [116]. Nevertheless, the integrity of the membrane’s structure is only maintained at high temperatures (>790 °C), where operating at lower temperatures induces phrase transformation. Moreover, Pei et al. [117] investigated the stability of a SrCr_0.8_Fe_0.2_$O_{3-\delta }$ membrane. The study identified two potential factors that could contribute to the degradation of membrane stability and the occurrence of membrane cracking. These factors are the surface tension force and the mismatch in the thermal expansion coefficient.

By introducing partial substitutions of other metal ions into the SrCr_0.8_Fe_0.2_$O_{3-\delta }$ perovskite membrane, the stability can be effectively enhanced. For instance, the doping of La^3+^ in a perovskite structure of La_1−x_SrCr_x_Fe_0.2_$O_{3-\delta }$ resulted in a material that is stable and resistant to inert nitrogen [118]. Substitution of A-site cation in the perovskite oxide structure yielded improved O_2_ permeability and structural stability. For example, replacing Sr^2+^ with Ba^2+^ in a Ba_0.5_Sr_0.5_Co_0.8_Fe_0.2_$O_{3-\delta }\;$perovskite oxide membrane [119]. Other strategies involved the B-site cation substitution of the perovskite oxide in order to enhance the stability of OTMs subjected to extreme operating conditions. For example, replacing cobalt ions with Ga^3+^, Ti^4+,^ and Zr^4+^ in a structure of BaSr_0.2_Co_0.4_Fe_0.4_$O_{3-\delta }$ yieleded stable O_2_ flux for 2200 h at an elevated temperature of 850 °C [120,121]. 

Despite significant advancements in increasing oxygen flux and stability, there are still numerous challenges that need to be addressed to enable the practical application of perovskite OTMs. One such challenge is the difficulty in finding a single perovskite membrane material that exhibits both high oxygen permeation fluxes and meets the requirements for industrial applications. However, in the bi-phase membrane technique, the dispute of reduced electronic conductivity in the highly stable phase, which has low oxygen permeability, can be resolved by adding an additional phase with higher electronic conductivity. For instance, a bi-phase membrane was fabricated using an MIEC Ce_0.85_Gd_0.1_Cu_0.02_$O_{2-\delta }$ and electronic conducting MIEC La_0.6_Ca_0.4_Fe$O_{3-\delta }$. This approach allows instantaneous ionic and electronic O_2_ transport in both MIEC phases. The highest O_2_ flux of 0.70 mL cm^−2^ min^−1^ was achieved using a membrane with a thickness of 0.5 mm. The operating temperature was set at 950 °C, and CO_2_ was utilized as the sweep gas [122]. Most of the research efforts in this field have primarily concentrated on traditional disk-shaped membranes as they can be easily fabricated using conventional and facile techniques such as static pressing. However, disk membranes possess an extremely limited membrane area, which poses challenges when applied in multiple planar stack configurations, including issues related to sealing, connection, and pressure resistance. The O_2_ separation is enhanced with the increase in area per unit volume of membranes, which can be achieved through alternative geometries such as thin tubes and hallow fibers [123,124]. Hollow fiber perovskite membranes have demonstrated remarkable O_2_ permeation fluxes exceeding 10 mL cm^−2^ min^−1^ [125,126]. Nevertheless, these membranes may not be economically viable for long-term industrial applications due to their performance deterioration over time. Another approach focuses on utilizing highly stable materials coupled with surface engineering to improve O_2_ permeation flux and membrane stability [127]. These challenges need to be overcome to realize the widespread practical utilization of perovskite mixed conducting membranes. Table 7 summarizes the oxygen permeation fluxes of selected perovskite OTMs.

## 5. Perovskite Membranes in Carbon Dioxide Capture

**Figure 5:**
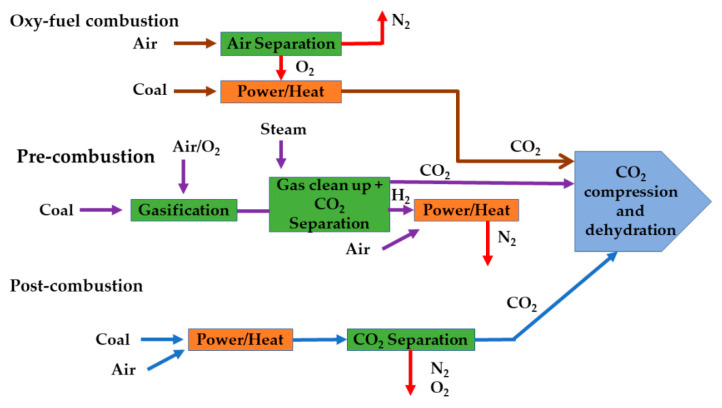
Schematic of carbon dioxide capturing technologies.

Reducing carbon dioxide (CO_2_) emissions is crucial for mitigating the impacts of climate change and transitioning towards a more sustainable future. CO_2_ capture technologies are utilized to reduce emissions. These technologies include pre-combustion, post-combustion, and oxy-fuel combustion [137,138]. These methods are highlighted in Figure 5. Pre-combustion is primarily employed in coal gasification plants, while both post-combustion and oxy-fuel combustion can be applied to both coal- and gas-fired plants, with post-combustion considered to be the most mature compared to its counterparts [139]. In oxy-fuel combustion, the fuel is burned using pure oxygen rather than atmospheric air. This approach significantly removes nitrogen in the exhaust gas, which has an impact on the successive separation process. Moreover, the technology provides a significant reduction in NOx emissions [140]. Cryogenic air separation is currently the standard process used in oxy-fuel power plants. Nevertheless, due to excessive energy consumption, a reduction in the overall plant efficiency by 10–12% is reported [141]. Perovskite-based OTMs are considered the most favorable substitutes for conventional methods.

**Figure 6:**
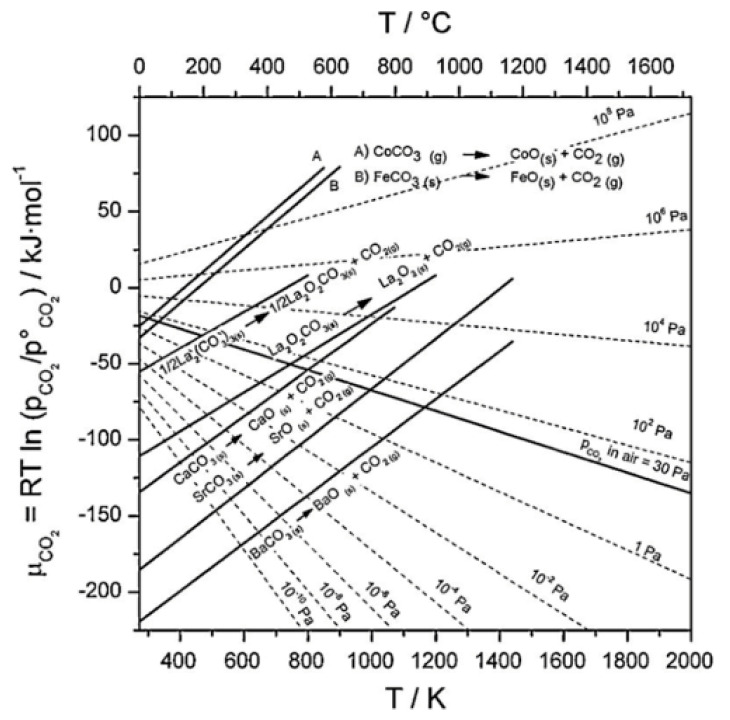
Ellingham diagram for various carbonates [152]. (Obtained from an open access source).

Important factors are required for the application of perovskite-based membranes in the oxy-fuel combustion process. For instance, having adequate O_2_ permeability while maintaining long-term stability under CO_2_ atmosphere [142]. The main issue with perovskite membranes is the presence of alkaline-earth elements such as Ca, Sr, or Ba. When exposed to environments with CO_2_ gas, these elements form a carbonate layer that impedes the diffusion of O_2_ into the membrane, leading to a diminished flux. The CO_2_ resistance of materials containing alkaline-earth elements can be theoretically evaluated with the aid of the Ellingham diagram [143]. The resistance is evaluated at a certain temperature and partial pressure. In the diagram (seen in Figure 6 [144]), the dashed lines on the diagram represent the chemical potential of CO_2_ at various CO_2_ partial pressures. The solid continuous lines represent the decomposition potential of specific carbonates. To assess the CO_2_ resistance, compare the position of the CO_2_ chemical potential line with the carbonate decomposition line. If the CO_2_ chemical potential line lies below the decomposition line, it suggests that the carbonate is thermodynamically unstable and may decompose.

Materials with high O_2_ permeability, such as SrCo_0.8_Fe_0.2_$O_{3-\delta }$, demonstrated a significant decrease in flux when subjected to CO_2_ environment [145]. In order to improve the CO_2_ resistance of such membranes, various strategies have been employed, including the partial substitution of Sr^2+^ with La^3+^ [146], or Co/Fe with Ti^4+^ [145], Zr^4+^ [147], Ta^5+^ [148], or Nb^5+^ [149]. However, this increase in CO_2_ resistance is inversely proportional to the O_2_ flux [150]. In addition to site substitution, the utilization of bi-phase OTMs has been explored to improve CO_2_ resistance, in spite of their low O_2_ permeabilities [151,152]. Another method is to increase the O_2_ partial pressure in CO_2_-dominant environment, where it was reported that the CO_2_ resistance is enhanced with the rise in O_2_ partial pressure [150,152]. For instance, a membrane maintained a high O_2_ permeability of 0.84 mL cm^−2^ min^−1^ when the partial pressure of O_2_ was increased [152]. The impact of sulfur dioxide (SO_2_), which is a by-product of flue gas in power plants, on the O_2_ permeation of perovskite materials has been examined [153]. It was stated that the oxygen permeability of La_0.6_Sr_0.4_Co_0.2_Fe_0.8_$O_{3-\delta }$ hollow fiber membranes were diminished when a small quantity of SO_2_ was added to the sweep gas, and this poisoning effect was irreversible [80]. The exposure to SO_2_ resulted in the decomposition of the La_0.6_Sr_0.4_Co_0.2_Fe_0.8_$O_{3-\delta }$ material and the formation of SrSO_4_, causing severe damage to the membrane.

## 6. Challenges, Limitations, and Future Work

### 6.1. Membranes for Hydrogen Production

The majority of the reported perovskite membranes yield H_2_ permeation flux lower than the necessitated standard of 1–2 mL cm^−2^ min^−1^ at temperatures ranging from 600–700 °C for commercial applications. Additionally, there is a significant variation in the reported hydrogen flux performances, even when considering membranes with the same composition. This variation can be attributed to differences in synthesis, fabrication, and deposition methods as well as test conditions. Furthermore, variations in membrane microstructures resulting from different preparation methods can influence both protonic and electronic conductivities. Under harsh operating conditions, such as elevated temperatures, the reactivity of perovskite-based proton conducting membranes with the sweep gas species (such as water vapor, CO_2_, etc.) intensifies. This heightened reactivity can result in a decrease in the mechanical, chemical, and structural stability of the perovskite-based membranes, leading to impulsive degradation in flux performance. Despite significant advancements in membrane materials and performance, achieving an ideal membrane that possesses both high H_2_ permeation flux and excellent stability simultaneously remains challenging.

The primary focus of research on perovskite-based membranes has been on the development of novel composites that offer improved H_2_ permeation flux and stability. However, research has not focused on the cost effectiveness of the said composites. Existing processing technologies have certain constraints, including challenges in complex powder synthesis and achieving consistent reproducibility at a medium to low level. It is crucial to conduct further assessments on the cost-effectiveness and environmental aspects associated with the current perovskite membrane materials and methods in order to detect the main factors driving the cost.

### 6.2. Membranes for Oxygen Separation

The primary challenges in the development and commercial implementation of OTMs mainly revolve around fabrication, ensuring long-term reliable operation, and integration of robust systems. It is crucial to minimize material degradation caused by interactions between gases and solids as well as between different solid components. Furthermore, it is essential to develop a thorough comprehension of transport kinetics and establish universally accepted protocols for assessing oxygen flux and permeation. There are substantial gaps in knowledge within these domains, necessitating further investigation and research efforts.

Based on the aforementioned discussions, it can be recommended that A-site cation of the perovskite structure can be doped by strontium, while B-site cation can be doped by Mn, Ni, and Fe to realize the required properties concurrently. Specifically, the La_0.7_Sr_0.3_Cr_0.7_Ti_0.3_$O_{3-\delta }$ composition based on titanium exhibits the lowest oxygen nonstoichiometric. Overall, the composition La_1−x_Sr_x_Cr_1−y_M_y_Ti_z_O_3_ would be the most promising perovskite composition for OTMs. To achieve a better O_2_ flux and performance, combining the perovskite phase with the fluorite phase, such as 8YSZ (8 mol% yttria-stabilized zirconia), is recommended to enhance ionic conductivity. Further fundamental research is required in the areas of transport behavior and electrochemical activity, particularly for doped lanthanum chromites, to fully exploit their potential for active OTM application.

### 6.3. Membranes for Carbon Dioxide Capture

**Figure 7:**
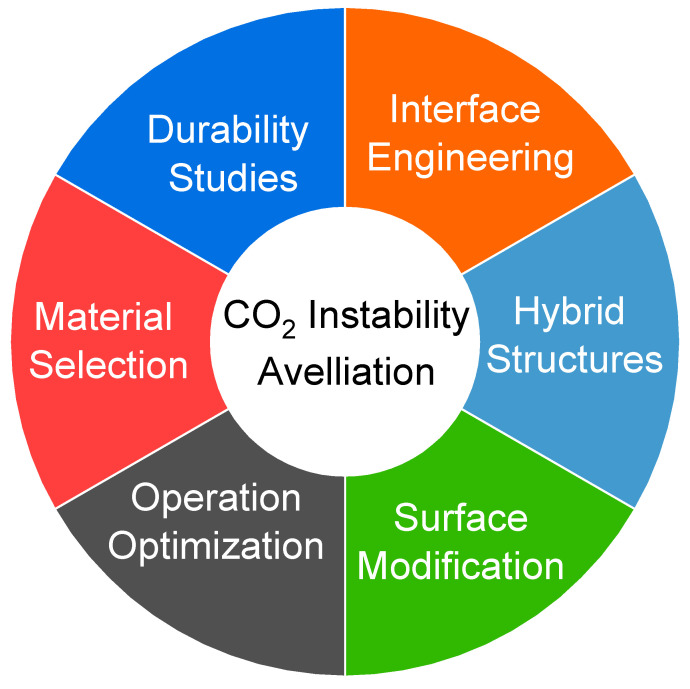
Strategies to alleviate the CO_2_ capture through perovskite membranes.

Perovskite membranes have shown potential for CO_2_ capture, but several challenges need to be addressed. One major challenge is the stability of perovskite materials subjected to CO_2_ as it can cause structural degradation and reduced performance. Achieving high selectivity for CO_2_ over other gases is also challenging. Perovskite membranes often exhibit lower permeability and flux compared to other materials, necessitating improvements in gas transport properties. Nevertheless, various strategies can be implemented to alleviate this instability and improve the performance and durability of perovskite membranes in CO_2_ separation. These strategies are highlighted in Figure 7.

The choice of perovskite material composition plays an integral part in enhancing the stability of the membrane in CO_2_ gas separation. Certain elements or combinations of elements can provide improved chemical stability and resistance to CO_2_ exposure. For example, incorporating elements with high oxygen affinity or resistance to carbonate formation can mitigate degradation caused by CO_2_. Surface modifications and coatings can be applied to perovskite membranes to enhance their stability in CO_2_ gas separation. These coatings can act as protective layers, preventing direct contact between the perovskite material and CO_2_. For instance, thin oxide films or dense ceramic coatings can provide a barrier against CO_2_ attack and improve the membrane’s resistance to degradation. Combining perovskite materials with other stable and compatible materials in composite or hybrid structures can help alleviate the instability in CO_2_ gas separation. By integrating a stable support or protective layer with the perovskite membrane, the overall stability and durability can be improved. This approach can enhance the resistance to CO_2_-induced degradation and prolong the membrane’s lifespan. Adjusting the operating conditions, such as temperature, pressure, and gas composition, can help alleviate the instability of perovskite membranes in CO_2_ gas separation. Optimizing these parameters can minimize the adverse effects of CO_2_ exposure on the perovskite material. Additionally, optimizing the gas composition, such as controlling the presence of impurities or reactive species, can mitigate the degradation caused by CO_2_. The interface between the perovskite membrane and the surrounding environment can be engineered to improve stability. This includes designing compatible interlayers or coatings that offer chemical resistance and prevent CO_2_-induced degradation. Surface functionalization methods can also be utilized to improve the stability and performance of the perovskite membrane. Conducting long-term durability studies under realistic operating conditions is crucial to understanding and mitigating the instability of perovskite membranes in CO_2_ gas separation. By monitoring the membrane’s performance over extended periods and identifying degradation mechanisms, researchers can develop strategies to enhance stability and design more robust membranes.

## 7. Conclusions

In conclusion, a comprehensive overview of the progress, challenges, and potential applications of perovskite membranes in various gas production, separation, and capture processes is presented. The paper highlights the promising features of perovskite-based membranes, including their high proton conductivity, oxygen permeation capabilities, and potential for use in hydrogen production and carbon capture technologies.

Advancements in perovskite membrane research have focused on enhancing their performance by optimizing composition, microstructure, and doping strategies. The development of novel perovskite compositions and exploration of dual-phase and hybrid materials have shown promise in achieving improved gas separation efficiency and stability. Furthermore, various strategies such as interface engineering and thin film fabrication techniques have been employed to enhance membrane performance. On the other hand, the review also emphasizes the existing challenges that need to be addressed for the widespread implementation of perovskite membranes. These challenges include improving chemical stability and long-term durability and reducing manufacturing costs. Additionally, the standardization of characterization techniques and evaluation methods is crucial for accurately assessing membrane performance and facilitating comparison among different studies.

## Figures and Tables

**Table 1:** Comparison between various membrane materials.

Aspect	Perovskite	Carbon	MOFs	COFs	Zeolites
Porosity/Surface Area	Moderate	High	High	High	High
Selectivity	Moderate	Moderate	High	High	High
Mechanical Strength and Stability	High	Moderate/High	Low	Low	High
Scalability	Developing	Established	Challenging	Challenging	Developing
Cost	Moderate	Low	High	High	Moderate

**Table 2:** A summary of the perovskite membrane fabrication techniques.

Fabrication Method	Advantages	Drawbacks
Disk Membranes	Facile Preparation	Thick membranes
Tape Cast	Homogenous membrane structure and controlled film thickness	Low thickness results in brittle membranes
Extrusion	Fine control of the dimensions	Two sintering steps are required
Hollow Fibers	State-of-the-art membrane geometry	Brittle membranes with a small internal diameter that causes pressure loss
Asymmetric film on porous substrates	Use of porous substrates that enhances the mechanical stability	The expansion thermal coefficient between the substrate and material needs to match
Freeze-cast	Mechanically stable substrates and an environmentally friendly process due to the use of water as a solvent	The cooling rate needs to be controlled, and post-fabrication treatments are required

**Table 3:** Summary of the H_2_ flux permeation for single-phase disk-shaped perovskite membranes.

Material	H_2_ Flux (mL cm^−2^min^−1^)	Thickness (mm)	Feed/Sweep Atmosphere	Ref.
SrCe_0.95_Tb_0.05_$O_{3-\delta }$	0.014	1.00	20% H_2_ + 80%He/0.001 atm CO + Ar	[61]
SrCe_0.95_Tb_0.05_$O_{3-\delta }$	0.016	1.00	20% H_2_ + 80%He/0.1 atm CO + Ar	[61]
SrCe_0.95_Tm_0.05_$O_{3-\delta }$	0.04	1.60	10% H_2_ + 90%He/air	[74]
SrCe_0.95_Tm_0.05_$O_{3-\delta }$	0.0425	1.60	10% H_2_ + 90%N_2_/20% O_2_ +80% Ar	[61]
SrCe_0.95_Tm_0.05_$O_{3-\delta }$	0.022	0.70	H_2_/Ar	[75]
SrCe_0.95_Tm_0.05_$O_{3-\delta }$	0.054	1.20	40% H_2_ + 50% He/Ar	[71]
SrCe_0.85_In_0.1_Tm_0.05_$O_{3-\delta }$	0.033–0.042	1.20	50% H_2_ + 50% He/Ar	[71]
SrCe_0.95_Zr_0.1_Tm_0.05_$O_{3-\delta }$	0.015	1.60	10% H_2_ + 90%N_2_/20% O_2_ + 80% Ar	[61]
SrCe_0.75_Zr_0.2_Tm_0.05_$O_{3-\delta }$	0.042	1.20	40% H_2_ + 60% He/Ar	[76]
SrCe_0.95_Yb_0.05_$O_{3-\delta }$	0.019	0.70	H_2_/Ar	[75]
BaCe_0.8_Yb_0.2_$O_{3-\delta }$	0.01	1.00	50% H_2_ + 50% He/N_2_	[77]
BaZr_0.9_Fe_0.1_$O_{3-\delta }$	0.75	1.15	20% H_2_ + 80% He/Ar	[72]
BaCe_0.95_Nd_0.05_$O_{3-\delta }$	0.017–0.026	0.70	80% H_2_ + 20%H_2_/98% Ar + 2% N_2_	[73]

**Table 4:** Summary of the H_2_ flux permeation for double-phase disk-shaped perovskite membranes.

Material	Ratio	H_2_ Flux (mL cm^−2^min^−1^)	Thickness (mm)	Feed/Sweep Atmosphere	Type	Ref.
Pd-CaZr_0.9_Y_0.1_$O_{3-\delta }$	50:50 vol%	1.30–2.30	0.55	20% H_2_ + 80% He/Dry N_2_	Cermet	[85]
Pd-CaZr_0.9_Y_0.1_$O_{3-\delta }$	50:50 vol%	0.89	0.47	20% H_2_ + 30% CO_2_ + 50% He/Dry N_2_		[85]
Pd-BaCe_0.4_Zr_0.4_Gd_0.1_DY_0.1_$O_{3-\delta }$	50:50 vol%	2.77	0.40	100% H_2_ /Dry N_2_		[78]
Pd-BaCe_0.4_Zr_0.4_Gd_0.1_DY_0.1_$O_{3-\delta }$	50:50 vol%	1.25	0.40	50% H_2_ + 50% CO_2_ /Dry N_2_		[78]
Ni-BaCe_0.85_Tb_0.05_Zr_0.4_$O_{3-\delta }$	50:50 wt%	0.17	0.50	Dry 50% H_2_ + 50% He/Ar		[86]
Ni-BaCe_0.9_Y_0.1_$O_{3-\delta }$	40:60 vol%	0.76	0.40	Dry 4% H_2_ + 96% He/N_2_ +H_2_		[81]
Ni-BaZr_0.1_Ce_0.7_Y_0.2_$O_{3-\delta }$	40:60 vol%	0.056	1.0	Wet 4% H_2_ + 96% He/N_2_ +H_2_		[79]
Ni-BaZr_0.1_Ce_0.7_Y_0.2_$O_{3-\delta }$	40:60 vol%	0.15	0.75	Wet 40% H_2_ + 20% CO_2_ + 40% He/N_2_ +H_2_		[87]
Ni-BaZr_0.1_Ce_0.7_Y_0.2_$O_{3-\delta }$	40:60 vol%	0.805	0.266	100% H_2_/He		[79]
Ni-BaZr_0.1_Ce_0.7_Y_0.2_$O_{3-\delta }$	30:70 vol%	0.0336	0.5	40% H_2_ + 58.5% N_2_ + 1.5% H_2_O/Ar		[88]
Ni-BaZr_0.1_Ce_0.7_Y_0.2_$O_{3-\delta }$	30:70 vol%	0.0268	0.5	40% H_2_ + 1.5% H_2_O + N_2_ + 30 ppm H_2_S/Ar		[88]
Ni-BaZr_0.1_Ce_0.7_Y_0.1_Yb_0.1_$O_{3-\delta }$	40:60 vol%	0.0174	0.75	20% H_2_ + 80% He/N_2_		[89]
Ni-BaZr_0.1_Ce_0.7_Y_0.1_Yb_0.1_$O_{3-\delta }$	40:60 vol%	0.0456	0.75	20% H_2_ + 60% CO_2_ + 20% He/N_2_		[89]
Ni-BaZr_0.1_Ce_0.7_Y_0.1_Yb_0.1_$O_{3-\delta }$	40:60 vol%	0.118	0.56	40% H_2_ + 57% He + 3% H_2_O/N_2_		[90]
Ni-BaZr_0.1_Ce_0.7_Y_0.1_Yb_0.1_$O_{3-\delta }$	40:60 vol%	0.127	0.56	40% H_2_ + 10% CO_2_ + 47% He + 3% H_2_O/N_2_		[90]
BaCe_0.2_Zr_0.7_Y_0.1_$O_{3-\delta }$-Sr_0.95_Ti_0.9_Nb_0.1_$O_{3-\delta }$	50:50 vol%	0.011	1	Dry 4% H_2_ +96% He/dry Ar	CerCer	[91]
BaCe_0.8_Zr_0.2_$O_{3-\delta }$-Ce_0.8_Y_0.2_$O_{2-\delta }$	50:50 wt%	0.0744	1.44	Wet 50% H_2_ + He + Ar/Ar		[83]
BaCe_0.65_Zr_0.2_Y_0.15_$O_{3-\delta }$-Ce_0.85_Gd_0.15_$O_{2-\delta }$	50:50 vol%	0.27	0.65	Wet 50% H_2_ +50% He/Wet Ar		[92]
BaCe_0.65_Zr_0.2_Y_0.15_$O_{3-\delta }$-Ce_0.85_Gd_0.15_$O_{2-\delta }$	50:50 vol%	2.40	0.65	Dry 50% H_2_ +50% He/Wet Ar		[92]
BaCe_0.65_Zr_0.2_Y_0.15_$O_{3-\delta }$-Ce_0.85_Gd_0.15_$O_{2-\delta }$	60:40 vol%	0.14	0.65	Wet 50% H_2_ +50% He/Wet Ar		[92]
BaCe_0.65_Zr_0.2_Y_0.15_$O_{3-\delta }$-Ce_0.85_Y_0.15_$O_{2-\delta }$	50:50 vol%	0.12	0.61	Wet 50% H_2_ +50% He/Wet Ar		[92]
SrCe_0.95_y_0.05_$O_{3-\delta }$-Zno	90:10 wt%	0.039	1.09	21% H_2_ + 79% He/N_2_		[82]
La_5.5_W$O_{11.25-\delta }$-La_0.87_Sr_0.13_Cr$O_{3-\delta }$	50:50 vol%	0.15	0.37	Wet 50% H_2_ +50% He/Wet Ar		[84]

**Table 5:** Summary of the H_2_ flux permeation for asymmetric perovskite membranes.

Material	H_2_ Flux (mL cm^−2^min^−1^)	Thickness (mm)	Feed/Sweep Atmosphere	Ref.
SrCe_0.95_Tm_0.05_$O_{3-\delta }$	0.125	0.15	10% H_2_ + 90% He/Air	[100]
SrCe_0.95_Yb_0.05_$O_{3-\delta }$	0.013	0.02	H_2_ + He/N_2_ + O_2_ + He	[95]
SrCe_0.95_Zr_0.05_Eu_0.1_$O_{3-\delta }$	0.21–0.23	0.033	100% H_2_/He	[96]
BaCe_0.9_Y_0.1_$O_{3-\delta }$	0.113	0.01	Dry 10% H_2_ + 90% N_2_/Ar	[93]
BaCe_0.9_Y_0.1_$O_{3-\delta }$-Zr	0.17	0.01	Dry 10% H_2_ + 90% N_2_/Ar	[93]
BaCe_0.85_Tb_0.05_Zr_0.1_$O_{3-\delta }$	0.07	0.05	50% H_2_ + 50% He/Air	[94]
Ni-BaCe_0.95_Tb_0.05_$O_{3-\delta }$	0.914	0.09	50% H_2_ + 50% N_2_/He	[98]
Ni-BaZr_0.1_Ce_0.7_Y_0.2_$O_{3-\delta }$	0.32	0.003	80% H_2_ + 17% He + 3% H_2_O/Dry Ar	[101]
Ni-BaZr_0.1_Ce_0.7_Y_0.1_Yb_0.1_$O_{3-\delta }$	0.49	0.044	Dry 100% H_2_/He	[99]
Ni-BaZr_0.1_Ce_0.7_Y_0.1_Yb_0.1_$O_{3-\delta }$	1.12	0.044	Dry 100% H_2_/He	[99]

**Table 6:** Summary of the H_2_ flux permeation for hollow fiber perovskite membranes.

Material	H_2_ Flux (mL cm^−2^min^−1^)	Thickness (mm)	Feed/Sweep Atmosphere	Ref.
SrCe_0.95_Yb_0.05_$O_{3-\delta }$	0.2	-	35.9% H_2_ + 64.1% Ar/Dry Air	[107]
BaCe_0.8_Y_0.2_$O_{3-\delta }$	0.38	0.20	50% H_2_ + 50% He/N_2_	[77]
BaCe_0.95_Tb_0.05_$O_{3-\delta }$	0.57	0.10	50% H_2_ + 50% He/N_2_	[102]
BaCe_0.85_Tb_0.05_Co_0.1_$O_{3-\delta }$	0.385	0.163	50% H_2_ + 50% He/N_2_	[103]
NiO-BaCe_0.95_Tb_0.05_$O_{3-\delta }$	0.53	0.015	50% H_2_ + 50% He/He	[104]
BaCe_0.85_Tb_0.05_Co_0.1_$O_{3-\delta }$	0.009	0.132	50% H_2_ + 50% N_2_/He	[105]
BaCe_0.85_Tb_0.05_Co_0.1_$O_{3-\delta }$	0.164	0.132	50% H_2_ + 50% N_2_/He	[105]
BaCe_0.95_Tb_0.05_$O_{3-\delta }$	0.046–0.272	0.10	50% H_2_ + 50% N_2_/He	[106]

**Table 7:** Oxygen permeation flux for various perovskite-based oxygen transport membranes.

Material	H_2_ Flux (mL cm^−2^ min^−1^)	Thickness (mm)	Ref.
SrCo_0.8_Sc_0.2_$O_{3-\delta }$	3.09	1	[128]
SrCo_0.9_Ti_0.1_$O_{3-\delta }$	1.82	0.65	[129]
SrCo_0.9_Nb_0.1_$O_{3-\delta }$	4.24	1	[115]
SrCo_0.8_Fe_0.2_$O_{3-\delta }$	0.23	1	[130]
BaBi_0.4_Co_0.2_Fe_0.4_$O_{3-\delta }$	0.80	1.5	[131]
BaCe_0.15_Fe_0.85_$O_{3-\delta }$	0.42	1	[121]
Ba_0.5_Sr_0.5_Co_0.8_Fe_0.2_$O_{3-\delta }$	2.10	1.8	[119]
Ba_0.5_Sr_0.5_Co_0.8_Fe_0.2_$O_{3-\delta }$	4.39	0.22	[132]
Ba_0.5_Sr_0.5_Zn_0.2_Fe_0.8_$O_{3-\delta }$	0.34	1.45	[133]
La_0.6_Sr_0.4_Co$O_{3-\delta }$	1.03	1.5	[115]
LaGa_0.5_Ni_0.5_$O_{3-\delta }$	0.08	1	[134]
La_0.6_Sr_0.4_Co_0.2_Fe_0.8_$O_{3-\delta }$	0.03	1	[114]
La_0.6_Sr_0.4_Co_0.2_Mn_0.8_$O_{3-\delta }$	0.49	1.5	[115]
La_0.6_Sr_0.4_Co_0.8_Ni_0.2_$O_{3-\delta }$	1.44	1.5	[115]
La_0.8_Sr_0.2_Co_0.3_Ga_0.7_$O_{3-\delta }$	0.31	0.5	[135]
La_0.8_Sr_0.2_Fe_0.3_Ga_0.7_$O_{3-\delta }$	0.58	0.5	[136]
La_0.8_Sr_0.2_Ni_0.3_Ga_0.7_$O_{3-\delta }$	0.35	0.5	[136]

## Data Availability

Not Applicable.
